# School characteristics and pupils’ thoughts of leaving upper secondary school: The INSchool project

**DOI:** 10.3389/fpsyg.2024.1270139

**Published:** 2024-02-15

**Authors:** Emilie Aas Torland, Cecilie Karlstad, Mikkel Magnus Thørrisen, Tore Bonsaksen, Sturla Inge Haslerud, Randi Wågø Aas

**Affiliations:** ^1^Mestringsenheten (The Coping Unit), Department of Mental Health, Municipality of Sandnes, Sandnes, Norway; ^2^School Health Services, Municipality of Sola, Sola, Norway; ^3^Department of Rehabilitation Science and Health Technology, Faculty of Health Sciences, Oslo Metropolitan University, Oslo, Norway; ^4^Department of Public Health, Faculty of Health Sciences, University of Stavanger, Stavanger, Norway; ^5^Department of Health and Nursing Sciences, Faculty of Social and Health Sciences, Inland Norway University of Applied Sciences, Elverum, Norway; ^6^Department of Health, Faculty of Health Studies, VID Specialized University, Stavanger, Norway

**Keywords:** school participation, school dropout, school demands, decision control, social support, upper secondary school

## Abstract

**Introduction:**

Understanding pupils’ thoughts about leaving school may contribute to better identify those at risk of dropping out. Thus, we explored the associations between perceived psychological demands, decision control, and social support from teacher and fellow pupils, and pupils’ thoughts about leaving upper secondary school.

**Methods:**

Cross-sectional data from a convenience non-probability sample of 249 pupils from 12 Norwegian upper secondary schools were collected using a school-modified version of the work-focused Job Content Questionnaire (JCQ). Adjusted logistic regression was used to analyze the data.

**Results:**

Pupils who experienced higher psychological demands and lower social support from fellow pupils were more likely to experience thoughts of leaving school compared to those who experienced lower demands and high levels of social support. Decision control was not significantly associated with thoughts about leaving school.

**Conclusion:**

High psychological demands may increase the likelihood of considering leaving school. Peer support can lessen such thoughts. *Implication*: Identifying whether pupils are thinking about leaving school can help identify those who are at risk of dropping out of upper secondary school.

## Introduction

Several studies have investigated risk factors for dropping out of school. A review from Bowers et al. (2013) concluded that poor academic performance appeared to be the most important risk factor. Gubbels et al. (2019) identified 635 risk factors for dropping out in 33 studies, and a meta-analysis showed that having to repeat a year, low academic performance, and low IQ/learning difficulties were the most important risk factors. In addition, studies that show that adolescents who identify with school and experience academic and social engagement with support from parents and teachers are more likely to complete their education (Markussen, 2010; Fall and Roberts, 2012). Lee and Burkam (2003) investigated structural- and organizational factors that could explain pupils drop out of school. They found that teacher support and the number and types of programs offered by the school were of importance, but so was the size of the school. The connection between school size and drop-out rates were not linear, where the smallest rate was both in the smallest schools, but also in the medium and very large schools. Large schools had the highest dropout rate.

Some of those who drop out of school do just fine, but the general consequences of dropping out are many. Young people who drop out of school experience health decrements, are more likely to end up in prison, earn less than those who complete their education, and have an increased need for public financial support (Christle et al., 2007; Freudenberg and Ruglis, 2007). Dropping out of school is also associated with a wide range of public health challenges, as higher risk of obesity, smoking, low levels of physical activity and premature death (Freudenberg and Ruglis, 2007). Dropping out of school can lead to negative consequences later in life, at individual, school, and community level due to the increased risk of disability and unemployment (Christle et al., 2007; Freudenberg and Ruglis, 2007; Frostad et al., 2015; Ellingsen, 2021).

The complex and often non-linear correlation between, demands, stress or pressure, health issues, and participation challenges as drop out from school, and its sociodemographic sensitive and subjective nature, presents challenges in understanding social inclusion and occupational justice (Townsend and Wilcock, 2004; Whiteford et al., 2020). Previous research has shown that the odds of experiencing subjective health problems were around 50 per cent higher in school classes with high demands compared to low (Eriksson and Sellström, 2010). In a report on experiences, causes and prevalence of mental health problems among adolescents, the main finding was that particularly the demands placed on the students, was most closely linked to stress-related mental health issues (Eriksen et al., 2017). Research from Finland has linked the perception of high school demands to burnout (Salmela-Aro, 2017) and has adopted a model from occupational health research, the Job Demands-Resources model (JD-R), developed in 2001.

JD-R is based on and elaborates on the Demand Control Support (DCS) model, which was used for this study. The demand-control model is a widely used model for describing the impact of the psychosocial work environment on employee strain, health, learning, and development. Even though studied for many years, the connection between demands and decision control was first introduced by the work sociologist Robert Karasek in 1979 (Karasek, 1979). Psychological job demands refer to the work pressure and workload, whereas decision latitude or control is the breadths of skills usable in the job and the authority each worker has over making decisions. In JCD-model, it is proposed that the psychological demands interact with the degree of decision control, generating four distinctly kinds of psychosocial work experiences—also known as job types (Karasek and Theorell, 1990; Karasek et al., 1998). These are high-strain jobs (high demands + low control), low-strain jobs (low demands + high control) active jobs (high demands + high control), and passive jobs (low demands + low control). If the demands are perceived as high and the decision control is low, job strain occurs. If, on the other hand, high demands are combined with a high level of decision control, growth, motivation, and learning occurs. School is pupils’ “workplace” and the development of health issues and participation challenges in working life and school appear to have many common traits. It could therefore be argued that there is a need for more research in a school context using system-theoretical explanatory models from working life, as the JDC-model contribute to.

Pupils’ thoughts of leaving school are an indicator of whether pupils end up leaving before completing upper secondary education (Christle et al., 2007; Frostad et al., 2015). In the literature, dropping out of school is understood as a process rather than an outcome, and thoughts of leaving school may occur long before pupils actually leave school (Lee and Burkam, 2003; Haugan et al., 2019; Tvedt et al., 2021a,b). This means that it is important to not only study the pupil and the situation during the phase in which the pupil drops out, but also to understand the factors that contribute to the development of thoughts about leaving school (Lee and Burkam, 2003; Frostad et al., 2015).

Despite growing knowledge about the risk factors for dropping out of school and thoughts of leaving, there are few studies that look at multiple different risk factors in context and based on a theoretical conceptual framework (Cairns et al., 1989; Alexander et al., 1997; Christle et al., 2007; Freudenberg and Ruglis, 2007; Frostad et al., 2015). There are also a few studies that investigate both factors that can contribute to thoughts of leaving and factors that could buffer against such thoughts.

Although school drop-out is a problem in most countries, this study’s context is Norway. Nineteen percent of those aged between 25 and 34 years in Norway have not completed upper secondary education (OECD, 2018), and it has been estimated that society could save up to 9 billion NOK (765 mill. Euro) each year if the proportion of people completing upper secondary education was to increase by 10 percentage points (Falch et al., 2009). Well-being among Norwegian pupils has decreased annually since 2012 (Bakken, 2019). Reduced well-being and lack of motivation in school are important reasons for poor academic performance and may increase the risk of dropping out of upper secondary education (Norwegian Directorate of Health, 2015). The number of young people in Norway receiving disability benefits has reached an all-time high. As of March 2021, 21,000 young people between the ages of 18 and 29 years were in receipt of disability benefits, an increase of 580 young people in just one year (Ellingsen, 2021). Only one fifth of disabled people between the ages of 18 and 34 years have completed primary and lower secondary education (17.7%) or upper secondary education (2.4%) (Norway, 2019; Ellingsen, 2021).

The objective of this study was to explore whether pupils’ perceptions of demands, decision control and social support from teachers and fellow pupils were linked to thoughts of leaving school, and whether control and social support in school moderated the correlation between psychological demands and thoughts of leaving school. The purpose of the project is to raise hypotheses that might bring increased knowledge of how school dropout can be prevented.

## Materials and methods

### Design

In this hypothesis-generating cross-sectional study, which is part of the INSchool project, a non-probability sample of pupils (*n* = 249) from upper secondary school (n = 12) completed an electronic questionnaire.

### Setting

The overall aim of the INSchool project is to secure a better understanding of the process of drop-out from school and to enable new tools for better identify pupils in risk of dropping out on an early stage. In the INSchool-project, we apply insight from occupational health research among employees, to better understand pupils in the school setting. The purpose of the project is to enable early identification and prevention of pupils in risk of falling out of school. This article is from the work package II which is an exploratory and hypothesis generating study.

### Sample: schools and pupils

The sample consisted of pupils from upper secondary schools in Norway across year cohorts, educational programs, and specializations. The recruitment of schools were in line with a heterogeneous non-probability sampling strategy, as the aim of the study was to generate hypothesis – not to test predefined hypothesis on a representative population. The recruitment of schools was finished in March 2020 (and therefore not affected by Covid-19). Schools in various parts of the country were contacted and asked to participate in the project. We used four different strategies to reach as many schools as possible and establish the most appropriate sample: (1) via e-mail, (2) by telephone, (3) via social media, and (4) through presentations for teachers and employees. At least two of these four strategies were used in each school. Those that agreed to participate were asked to appoint a local study coordinator who would assist in the recruitment of pupils. The invitation from the researchers to participate were sent by the local study coordinator. Students could choose to participate independently and needed to be at least 16 years old to do so.

Table 1 give a description of the 249 pupil that participated in the study. Two third were females, and most were on their second school year. One third were attending vocational programs, and the rest were attending general educational studies.

**Table 1:** Description of pupils participating in the study.

	Thoughts of leaving school, % (n)				Total (*n* = 249)	Yes (*n* = 89)	No (n = 160)	*p*
Gender				0.66
Female	69.1 (172)	36.6 (63)	63.4 (109)	
Male	30.9 (77)	33.8 (26)	66.2 (51)	
School year				0.88
Year 1	32.1 (80)	33.8 (27)	66.3 (53)	
Year 2	42.2 (105)	35.2 (37)	64.8 (68)	
Year 3	24.1 (60)	38.3 (23)	61.7 (37)	
Supplementary program for general university and college admission	1.6 (4)	50.0 (2)	50.0 (2)	
Type of program				0.11
General studies	63.8 (159)	32.1 (51)	67.9 (108)	
Vocational programs	36.1 (90)	42.2 (38)	57.8 (52)	

Statistical tests are chi-square.

### Data collection

Data collection was conducted electronically through the school’s study coordinator, or a recruitment station set up in the hallway or schoolyard. *Part 1* of the questionnaire dealt with sociodemographic questions relating to gender, county, specialization, and educational program (program for general studies vs. vocational programs). *Part 2* of the questionnaire involved a modified questions of some constructs of the Job Content Questionnaire (JCQ) (Karasek et al., 1998), which is based on the Demand, Control, Support (DCS) model (Karasek, 1979), which is extensively used to explain occupational health including participation problems in working life. The three constructs, demands, control, and social support are for many years well investigated topics in the occupational health literature. JCQ is built on this research forefront, up until late 90ties (Karasek et al., 1998). In this study, we refer to the modified version of the JCQ as the School Content Questionnaire (SCQ), where the JCQ wording of items is only changed from employees to pupils, and from job to school settings (DiStefano et al., 2009). The SCQ included questions about: *Decision latitude/control*: skill discretion (6 items) and decision authority (3 items). Skill discretion is measured by items that assess the level of skills and creativity required in the school setting, and the flexibility permitted the pupil in deciding what skills to employ. Decision authority assesses the organizationally mediated possibilities for pupils to make decisions about their school situation. *Psychological demands* (9 items) relate to how hard the pupil work, organization constraints on task completion, and conflicting demands. *Social support* is concerning teachers support (5 items) and co-pupils support (6 items). Responses to the statements were answered using a four-point Likert scale, ranging from “strongly disagree” to “strongly agree.” Examples of questions include: “Schoolwork provides me with good opportunities to make my own choices” (decision control), “Schoolwork involves an unreasonably high workload” (demands), “My fellow pupils are interested in me” (fellow pupil support) and “My teacher is helpful” (teacher support).

Several of the subscales had low and variable internal consistency (Cronbach’s alpha ranging from 0.5 to 0.6) since individual questions showed low correlation with the scale as a whole. To produce weighted total scores from the SCQ, principal component analysis was used to extract a single principal component for each scale, with respective factor loadings weighted for each question, and to calculate the scale score using regression (Valås, 2001). The questions that showed low consistency with the scale as a whole, were assigned a low weight in the principal component analysis and therefore had limited impact on the scale score.

*Part 3* included questions/statements about “thoughts of leaving school.” The statements were developed in a study conducted by Frostad et al. (2015) and are based on qualitative research conducted by Vallerand et al. (1992) and Rice and Harris (2005) into how pupils develop thoughts of leaving school. We used the “Intention to leave” domain (8 items). The statements were answered using a six-point VAS scale, ranging from “1 – completely false” to “6 completely true.”

There was some content-related overlap, with some statements from “thoughts of leaving school” being semantically related to demands. We also wanted a clear measure for intentions to leave school, for which possible causes of the intentions had not been assumed. Based on this, the outcome variable (“Thoughts of leaving school”) was created and we included only the three statements that did not address causal factors. The following statements were included in this outcome variable: “I often think about leaving this school,” “I wonder whether there is any reason to continue school” and “I feel as though I am wasting my time by going to school.”

A new, dichotomous variable was created and set to 1 (yes) if the respondent answered 4, 5 or 6 to one or more of the questions, which was interpreted as the respondent thinking about leaving school, or 0 (no) if all answers were 1, 2 or 3.

### Ethics statement

The study was approved by the Norwegian Social Sciences Data Service (NSD, project number 60106/749429). Each participant provided written informed consent. Before analyzing the data, all informants were deidentified and given an ID. No reward for taking part in the study was offered to the schools, teachers, or pupils.

### Analyses

IBM’s SPSS Statistics Version 25 was used in the analysis process. Descriptive analyses were first performed with frequency distributions for each item from SCQ, as well as the calculated total scores. The values of the questions “Strongly disagree,” “Disagree,” “Agree” and “Strongly agree” were set to “Agree” and “Disagree,” since dichotomization is frequently used when JCQ is applied. This is due to the concept of the four job types; high-strain jobs, low-strain jobs, passive jobs, and active jobs in the JDS-model that combined demand and control scores (Karasek and Theorell, 1990; Karasek et al., 1998) and make it possible to compare four risk groups. Dichotomization by using cut off in a visual binding analysis is also recommended by Karasek, and the JCQ center.

Since the data was not normally distributed, we used non-parametric Spearman’s rho correlation analyses to investigate the relationship between the independent and dependent variables. Group differences in terms of gender, school year and type of program were analyzed using chi-square tests, while group differences in terms of continuous variables derived from SCQ were analyzed using Mann–Whitney U tests. In connection with the Mann–Whitney tests, Cohen’s *d* was used as the effect size, with *d* values of around 0.20, 0.50 and 0.80 interpreted as small, moderate, and large effects, respectively (Limone and Toto, 2022).

In adjusted analyses of associations, gender and educational program were included as control variables. Sequential binary logistic regression analysis was used with the dichotomized variable “Thoughts of leaving school” (0: no, 1: yes) as the dependent variable. In block 1, the control variables gender and educational program (vocational vs. general studies) were added. Psychological school demands from SCQ was added to block 2. Social support from teachers and fellow pupils were both added to block 3. In the final block, block 4, we examined possible moderating factors. Decision control was not added, as we choose to set a limit for being included (*p*-value of <0.2 in univariate analysis). The reason for this was to keep the statistical power as high as possible.

## Results

Table 1 shows that a higher percentage of pupils on a vocational programs (42.2%) experienced thoughts of leaving school compared to pupils in general studies programs (32.1%), but there were no differences related to gender, year, or type of education program.

Table 2 compares pupils who had thoughts of leaving school with pupils who did not have thoughts of leaving school. Pupils who had thoughts of leaving school reported higher psychological demands (Cohen’s *d =* 0.48) and less social support from fellow pupils (Cohen’s *d =* 0.59) and teachers (Cohen’s *d =* 0.48) compared to those who did not have such thoughts.

**Table 2:** Differences between those who had thought about leaving school vs. those who had not thought about leaving school.

	Thoughts of leaving: yes*	Thoughts of leaving: no*	Cohen’s *d*	*p*
1a. Skills discretion	−0.05 (1.24)	0.02 (0.85)	0,07	0.69
1b. Decision authority	0.00 (1.03)	−0.01 (0.99)	0.01	0.52
2. Psychological school demands	0.30 (0.95)	−0.17 (0.99)	0.48	<0.001
3a. Social support from teachers	−0.30 (1.14)	0.18 (0.86)	0.48	<0.01
3b. Co-pupil social support	−0.38 (1.12)	0.21 (0.87)	0.0.59	<0.001

* Average values (SD) for scale *z* scores. *p*-values are based on Mann–Whitney *U*-tests.

Table 3 shows the results relating to factors associated with thoughts of leaving school. The model in block 1 (gender, educational program) did not provide a significant explanation beyond the zero model. The model in block 2 (gender, educational program, psychological school demands) was statistically significant, and psychological school demands were associated with thoughts of leaving school (OR = 1.70, CI: 1.28–2.26, *p* < 0.001). This means that for each standard deviation (or *z*-score) increase in the scale score for psychological school demands, the probability of experiencing thoughts of leaving school increases by 70 percent.

**Table 3:** Adjusted associations between possible predictors and thoughts of leaving school.

Block	Variable	Cox-Snell *r*^2^ (Nagelkerke *r*^2^)	OR (95% CI)	*p*
1		0.011 (0.014)		0.27
	Gender		1.09 (0.62–1.93)	0.77
	Educational program		1.54 (0.90–2.63)	0.12
2		0.066 (0.091)		0.001
	Gender		0.87 (0.48–1.59)	0.66
	Educational program		1.73 (0.99–3.02)	0.06
	Psychological school demands		1.70 (1.28–2.26)	<0.001
3		0.147 (0.202)		<0.001
	Gender		0.97 (0.52–1.82)	0.93
	Educational program		1.75 (0.97–3.14)	0.06
	Psychological school demands		1.71 (1.26–2.31)	0.001
	Social support from fellow pupils		0.58 (0.41–0.81)	0.001
	Social support from teachers		0.78 (0.56–1.08)	0.13
4		0.157 (0.215)		<0.001
	Gender		1.06 (0.56–2.01)	0.87
	Educational program		1.70 (0.94–3.07)	0.08
	Psychological school demands		1.69 (1.05–2.72)	<0.05
	Social support from fellow pupils		0.52 (0.32–0.84)	<0.01
	Social support from teachers		0.74 (0.53–1.04)	0.08
	Psychological demands × decision control		1.10 (0.77–1.56)	0.61
	Psychological demands × social support from fellow pupils		0.98 (0.60–1.58)	0.92
	Social support from fellow pupils × decision control		1.24 (0.81–1.91)	0.32

^*^ Cox-Snell *r*^2^ and Nagelkerke *r*^2^ are measures for the proportion of variations in the outcome, that are explained using the independent variables included in the model. OR is the odds rate. 95% CI is the 95% confidence interval for OR. Variable coding: Male = 1, female = 2, general studies = 1, vocational program = 2. The variables included in interaction analyses are dichotomized using the median value. Participants with z scores that are exactly the median value have been included in the lowest categories (low psychological demands, low decision control, and low support from fellow pupils).

The model in Block 3 (gender, educational program, psychological school demands, social support from fellow pupils, social support from teachers) was also statistically significant. Psychological school demands remained a significant predictor for experiencing thoughts of leaving school (OR = 1.71, CI: 1.26–2.31, *p* < 0.001) and the same was the case for lack of social support from fellow pupils (OR = 0.58, CI: 0.41–0.81, *p* < 0.001). This means that, for each *z*-score increase in social support from fellow pupils, the probability of experiencing thoughts of leaving school would be reduced by 42 percent. Social support from teachers was not independently associated with thoughts of leaving school.

In Block 4, we examined whether the associations between psychological demands and thoughts of leaving school and between support from fellow pupils and thoughts of leaving school were dependent on other factors. None of the interaction terms were statistically significant. In other words, there was no evidence to suggest that the levels of social support and decision control moderated the relationship between psychological school demands and thoughts of leaving school, and the level of decision control also did not moderate the relationship between social support from fellow pupils and thoughts of leaving school.

## Discussion

### Main findings

The objective of this study was to examine whether pupils’ perception of demands, control and social support at school were associated with thoughts of leaving school, and whether a sense of control and social support in school moderated the correlation between psychological demands and thoughts of leaving. The main findings will be discussed here: (1) Higher psychological school demands and lower social support from fellow pupils were associated with thoughts of leaving school, indicating that high psychological demands may be a risk factor, while social support from fellow pupils may serve as a protective factor from such thoughts occurring, (2) support from teachers and decision control did not show significant associations with thoughts of leaving school in adjusted analyses and (3) support from fellow pupils did not moderate the association between psychological school demands and thoughts of leaving.

For the purposes of this study, demands refer to pupils’ thoughts about schoolwork both in and outside of school. Ungdata (Bakken, 2019) showed that a significant proportion of adolescents view homework as a socially imposed construct that intervenes with leisure time, and this can be perceived as negative stress (Bakken, 2019) (Ungdata is a national data collection scheme, designed to conduct youth surveys at the municipal level in Norway). Haugan et al. (2019) examined to what extent a number of different protective factors could explain thoughts of leaving school. The factors they examined explained a total of 44.7 percent and the identified protective factors were parental support, teacher support, school commitment and the ability to handle stressful situations.

A number of previous studies have shown that a lack of support from teachers has been associated with thoughts of leaving school (Lessard et al., 2010; Frostad et al., 2015; Haugan et al., 2019; Thygesen et al., 2020; Tvedt et al., 2021a,b). We found similar trends in our study, with moderate correlations between teacher support and thoughts of leaving, but this was no longer significant in adjusted analyses. Teacher support has much more variation in the responses than fellow pupil support and the small number of participants in the study could explain why teacher support was not retained as a significant factor in adjusted analyses.

The study also found that pupils’ perception of decision control at school had no correlation with thoughts of leaving. This result contrasts with studies on pupils in higher education, in which pupils’ perception of independence and being able to make their own choices about conditions affecting their studies were associated with a higher degree of satisfaction (Haveraaen et al., 2017). Decision control has been shown to be important in a number of studies within occupational health research [see for example (Janssen et al., 2003)]. Compared to pupils in upper secondary school, pupils in higher education and employees may have a greater need for and expectations of agency in decision-making and this therefore is of greater importance among higher education pupils and adults than upper secondary school pupils. Decision control should therefore be examined further in new studies among pupils and should ideally be viewed in relation to other similar concepts from school research, such as the ability to handle stressful situations (Haugan et al., 2019).

Support from fellow pupils did not moderate the correlation between psychological school demands and thoughts of leaving. This does, to some extent, contrast with findings from occupational health research, in which high levels of social support have been found to provide a buffer against the negative impacts of high demands and little control of the job situation (Stevens, 1996).

### Methodological limitations

As this being an exploratory and hypothesis-generating study, a pre-registration of the study or any hypotheses were not relevant.

The sample was relatively small (*N* = 249), but was nevertheless statistically sufficient, as the number exceeded the required number pursuant to a recommended rate of 15 participants per predictor variable (Tabachnick and Fidell, 2013), as well as in relation to the formula *N* > 50 + (8 × number of predictors) (Norway, 2021). As we applied a non-probability sampling strategy, and the study was exploratory in nature we did not prioritize to get an overview of response rate and representativity of the sample. Still, to know more about possible limitations in views from the participants, it might be of interest to see how the sample was, compared to the population we studied. Based on figures from Statistics Norway (Rumberger, 1987), the variation between pupils on programs relating to general studies and vocational programs in the selection was not significantly different between the selection and the population (diff. = 1.8 percentage point, *p* = 0.559). However, compared to the population, girls were significantly overrepresented in the selection (diff. = 22.2 percentage points, *p* < 0.001). The study is based on a self-report questionnaire with its limitation. But the topic of interest in this study was about how the pupil experience their school situation – from their first-person point of view – which make PROM a relevant way to collect data. Further studies could extend the understanding by applying teachers and parents’ point of view and connect these self-report data to register data.

It is also a limitation for comparison purposes, that a payment to use the JCQ (to jcqcenter.com) has been required and paid. This may limit the use of JCQ in the future. Still, research about demand and control were widely explored and used when developing JCQ, and it is possible to assess the concepts similarly and comparably by using other instruments as the Copenhagen Psychosocial Questionnaire (COPSOQ) and the Nordic Ministries QPS-Nordic. In addition, assessing social support is the core construct in a dozen of different instruments. This might make it possible to compare the results from this study, also when other instruments are used to measure the constructs demand, control, and support.

Another weakness of the study was that we used a questionnaire that had not been validated for use with school pupils, even though it was based on one of the most commonly used and validated questionnaires for understanding dropout in working life. This challenge was reduced using principal component analyses to mitigate the effect of inconsistent individual questions in each subscale in the form. The exploratory nature of this study also made this a less dominant flaw.

In order to examine the causal relationships between demands, thoughts of leaving and actual dropouts from school, longitudinal studies will be required. To establish a good conceptual framework for understanding school dropouts, more studies using system-theoretical models and exploratory qualitative studies should be conducted.

### Implications for policy and practice

Dropping out of upper secondary school could lead to educational deficiencies on the part of the individual and this could lead to limitations on their financial and social situation throughout adulthood (6, 7, 9, 40). Having thoughts of leaving school may be a precursor to actually dropping out of school. The knowledge from this study therefore implies that reducing individually experienced negative demands or lack of social support could prevent vulnerable young people from dropping out of upper secondary school. Using decades of studying “drop-out from work” or prevention of work disability seems to give value for understanding the complex school drop-out problem. Hypotheses retrieved from this study requires more representative research, to verify our exploratory results. These results can also be used for development purposes. It challenges the need for more evidence-based tools for identifying those at risk. These hypotheses also call for innovation of preventive early preventive interventions, provided by a multidisciplinary School Health Services working close together with school management and teachers. These strategies align with the goals of the WHO initiative on health promoting schools, which seeks to create supportive living and learning environments that promote the physical, mental, emotional, and social wellbeing of students.

## Data availability statement

The original contributions presented in the study are included in the article/supplementary material, further inquiries can be directed to the corresponding author.

## Ethics statement

The study was approved by the Norwegian Social Sciences Data Service (NSD, project number 60106/749429). Each participant provided written informed consent.

## Author contributions

EAT: Data curation. Formal analysis. Writing – original draft. Validation. Writing – review & editing.CK: Data curation. Writing – review & editing. MMT: Data curation. Validation. Writing – review & editing. TB: Writing – review & editing. Validation. SiH: Writing – review & editing. RWA: Conseptualization. Team building. Project administration. Data curation. Validation. Formal analysis. Writing – original draft, Writing – review & editing.
